# Far rapid synthesis of giant DNA in the *Bacillus subtilis* genome by a conjugation transfer system

**DOI:** 10.1038/s41598-018-26987-0

**Published:** 2018-06-08

**Authors:** Mitsuhiro Itaya, Mitsuru Sato, Miki Hasegawa, Nobuaki Kono, Masaru Tomita, Shinya Kaneko

**Affiliations:** 1 Institute for Advanced Biosciences, Keio University, Nipponkoku, Daihoji, Tsuruoka-shi, Yamagata 997-0017 Japan; 2 School of Life Science and Technology, Tokyo Institute of Technology, 4259 Nagatsuta-cho, Midori-ku, Yokohama-shi, Kanagawa 226-8501 Japan

## Abstract

*Bacillus subtilis* offers a platform for giant DNA synthesis, which is mediated by the connection of overlapping DNA segments called domino DNA, in the cloning locus of the host. The domino method was successfully used to produce DNA fragments as large as 3500 kbp. However, domino DNA is limited to <100 kbp because of size restrictions regarding the transformation (TF) of *B. subtilis* competent cells. A novel conjugal transfer (CT) method was designed to eliminate the TF size limit. The CT method enables rapid and efficient domino reactions in addition to the transfer of giant DNA molecules of up to 875 kbp to another *B. subtilis* genome within 4 hours. The combined use of the TF and CT should enable significantly rapid giant DNA production.

## Introduction

**Figure 1:**
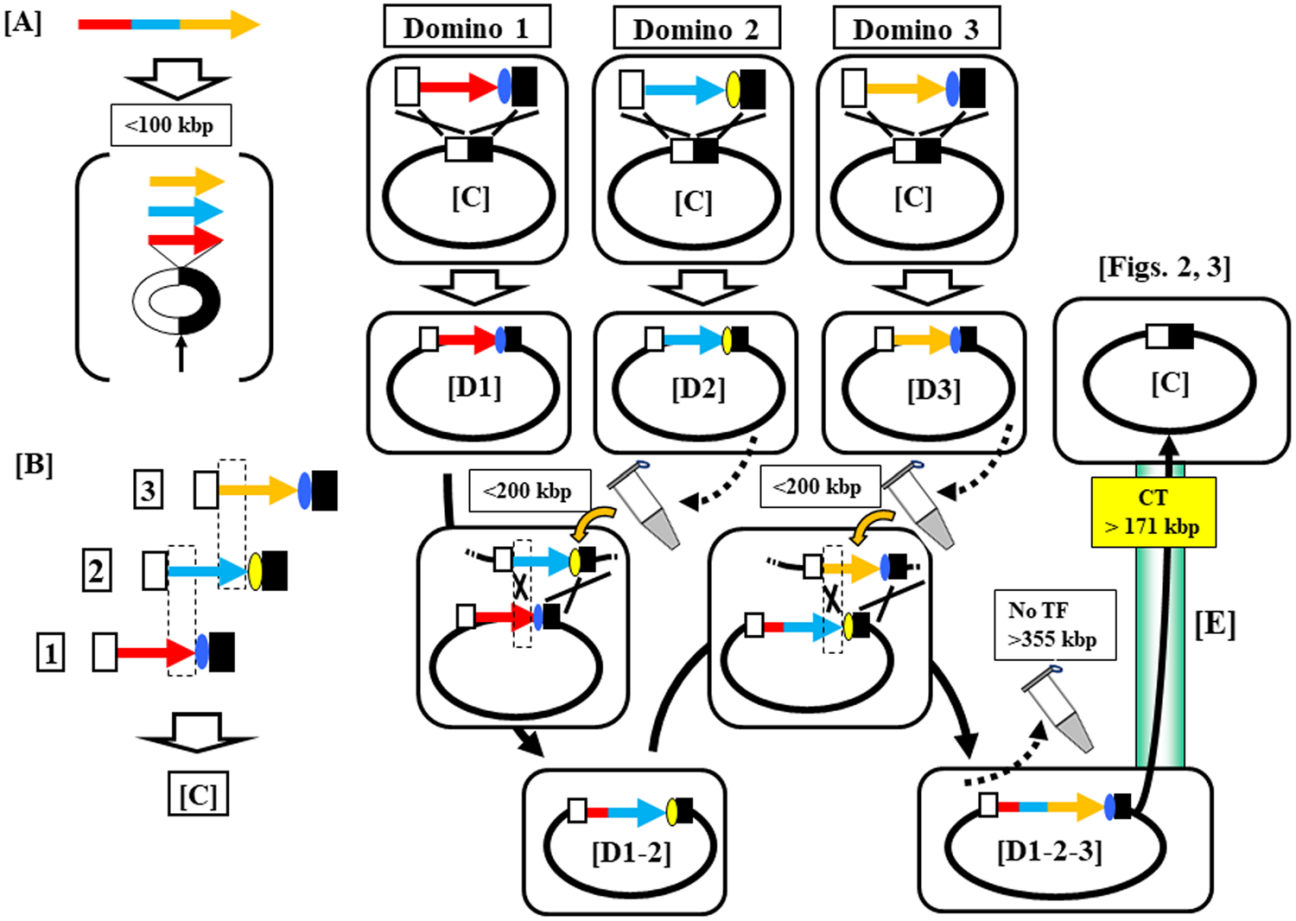
TF-mediated domino method in the *B. subtilis* genome requires liquid DNA and limits DNA size to <100 kbp per domino step. (**A**) The target DNA to be synthesized is divided into domino DNA. (**B**) Designed dominos possessing overlapping DNA sequences are prepared by the *E. coli* plasmids BAC^7,8^ or pBR322^1^ depending on the domino size. The size of dominos is restricted to <100 kbp even for BAC-based dominos. Dominos 1, 2, and 3 are shown in linearized form opened at the site of *E. coli* vectors, as indicated by the vertical arrow. Overlapping sequences between adjacent dominos are shown as rectangular dots. The red or blue ovals indicate antibiotic resistance genes conferring CmR or EmR, which are alternatively used for the selection of connected strains^1,2,8^. (**C**) The three domino DNA numbers are inserted in the *proB* locus by double homologous recombination between the *E. coli* vector sections shown by X, resulting in (D1) to (D3). Domino 2 elongates by double homologous recombination at the overlapping sequence, as shown by the rectangular dots between (D1) and (D2) DNA (the left X) and at the *E. coli* vector (right X). Similarly, Domino 3 integration is completed by the double homologous recombination sites. Note that selection markers are alternated at the elongation step. The small tube icon indicates genomic DNA prepared in liquid form for TF, indicating an average DNA size of <200 kbp. (**E**) The expected DNA transfer by the CT system of >171 kbp is indicated by solid arrows through a light green box. This is confirmed in Figs 2 and 3 and Table 1.

*Bacillus subtilis* Marburg 168, a Gram-positive spore-forming bacterium, is used as a platform for giant DNA synthesis^1–3^. The DNA synthesis is achieved by the connection of overlapping DNA molecules, called domino, at the cloning locus prepared in the *proB* gene locus of the *B. subtilis* genome, as illustrated in Fig. 1. The domino method has been used for the synthesis of various genomes, such as those of mouse mitochondria (16.7 kbp)^2^, lambda phage (48.5 kbp)^4^, rice chloroplast (135 kbp)^2^, and those of *Synechocystis* spp PCC6803 (3542 kbp)^1^. The domino method is based on the inherent natural transformation (TF), which is accurate enough to incorporate DNA^3^. Domino connection through TF requires liquid genomic DNA biochemically isolated from donor *B. subtilis*. As shown in Fig. 1, the liquid DNA is added to recipient competent cells. However, TF efficiency dramatically decreases with the increase in the size of the liquid DNA to >100 kbp^5–8^. Accordingly, our latest TF-based protocol restricts one domino size to ≤100 kb^8^.

We developed a conjugal transfer (CT) system for transmission of the *B. subtilis* genome to eliminate the TF-associated size restriction^9^. The CT enables the transfer of antibiotic resistance markers from the donor *B. subtilis* genome, distant from the origin of transfer *oriT*^110^ at the *xkdE* locus, to the recipient *B. subtilis* genome by mating within 4 hours at 30 °C^9^. Large DNA inserted in the *B. subtilis* genome should be similarly transferable with the help of a Type IV secretion system called T4SS, which is encoded and supplied by the plasmid pLS20*hyg*^9^. Orientation of the *oriT*^110^ from BEST390^9^ and in Table 1 was used throughout the study.

**Table 1:** *B. subtilis* strains used for TC and TF.

Size^a^	*B. subtilis* ^b^	Genotypes^b^	Antibiotic markers^c^	transcipients^d^	transformants^e^	Domino connection	
	RM125^9^	*leuB*8, *arg*-15, *hsd*RM					
	BEST386^9^	pHY300PLK	Tc				
5.7	BEST390^9^	x*kdE*::*oriT*^110^-*stm* unit	Sm		362 (SmR)		
	BEST23124^9^	pLS20*hyg*	Hm				
1.3	BEST4306^9^	*xkdE*::*oriT*^110^-*stm unit*, pLS20hyg, *yjcI*::*ne-spc*, *pycA*::*eo-bsr*	Sm Hm Spc BS	318			
						recipient *BEST6571*	recipient *BEST6568*
171	BEST6568^7^		Spc				
	*BEST6621*	*oriT*^110^-*stm*	Sm Spc		1 (SpcR)		
	*BEST6627*	*oriT*^110^-*stm*, pLS20*hyg*	Hm Sm Spc	176		23^f^	
196	BEST6571^7^		BS				
	*BEST6623*	*oriT*^110^-*stm*	Sm BS		18 (BSR)		
	*BEST6629*	*oriT*^110^-*stm*, pLS20*hyg*	Hm Sm BS	175			33^g^
345	BEST6586^7^		BS Spc				
	*BEST6625*	*oriT*^110^-*stm*	Sm BS Spc		0 (SpcR)		
	*BEST6631*	*oriT*^110^-*stm*, pLS20*hyg*	Hm Sm BS Spc	146			
509	BEST7095^1^		Sp Em				
	*BEST7817*	*oriT*^110^-*stm*	Sm Spc Em		0 (SpcR)		
	*BEST7817hyg*	*oriT*^110^-*stm*, pLS20*hyg*	Hm Sm Spc Em	125			
586	BEST7103^1^		Sp Em				
	*BEST7821*	*oriT*^110^-*stm*	Sm Spc Em		0 (SpcR)		
	*BEST7821hyg*	*oriT*^110^-*stm*, pLS20*hyg*	Hm Sm Spc Em	86			
683	BEST7118^1^		Sp Em				
	*BEST7823*	*oriT*^110^-*stm*	Sm Spc Em		0 (SpcR)		
	*BEST7823hyg*	*oriT*^110^-*stm*, pLS20*hyg*	Hm Sm Spc Em	140			
875	BEST7154^1^		Sp Em				
	*BEST7819*	*oriT*^110^-*stm*	Sm Spc Em		0 (SpcR)		
	*BEST7834*	*oriT*^110^-*stm*, pLS20*hyg*	Hm Sm Spc Em	3			

^a^Size of DNA in the *proB* gene, except BEST4305 and BEST390, which carry a blasticidin resistance gene (1.3 kbp) near *proB*^9^ and *oriT*^110^-*stm* unit (5.4 kbp) at the *xkdE*^9^, respectively.

^b^Previously published strains are shown with references in brackets. Strains constructed in this study are italicized. For strains referring^7^, their actual insert sizes are determined this study.

^c^Blasticidin S (Bs, 500 μg/mL), hygromycin (Hm, 150 μg/mL), spectinomycin (Spc, 100 μg/mL), streptomycin (Sm, 100 μg/mL), and tetracycline (Tc, 10 μg/mL).

^d^BEST386 (TcR) is used as the common recipient for all donors. Number of transcipients/mL in 4 hours by standard CT protocol^9^ selected by Spc and Tc, except for BEST6629 by BS and Tc.

^e^RM125 is used as the common recipient for TF with all genomic DNA. Volume of the competent cells is increased eight times that using standard transformation^1,5^. The antibiotics used for selection of the transformants are indicated in parentheses.

^f^A representative named CT[171/196] was analyzed by PFGE (Fig. 2) and sequenced.

^g^A representative named CT[196/171] was analyzed by PFGE (Fig. 2) and sequenced.

## Methods and Results

**Figure 2:**
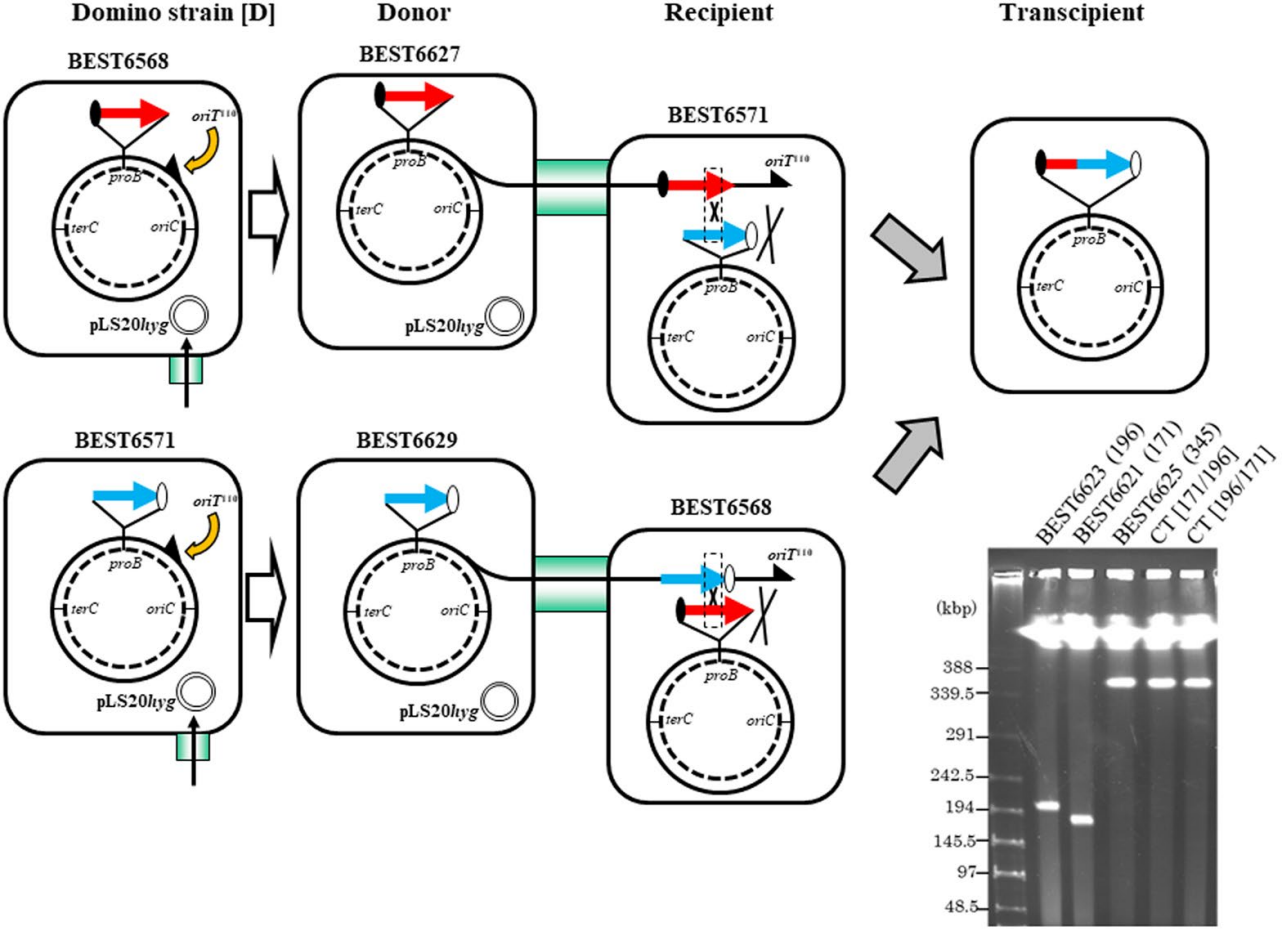
Domino connection by CT Top: The original BEST6568 was converted to donor BEST6627 by transfer of the *oriT*^110^ unit, as shown by a half triangle, and the helper plasmid pLS20*hyg*. Insertion of the *oriT*^110^ unit was achieved by TF from BEST390, as indicated by a curved yellow arrow. Introduction of pLS20*hyg* was mediated by CT from BEST23124, as indicated by a vertical arrow through a light green box. The donor BEST6627 carries a spectinomycin resistance gene (solid oval) located at the left end of the 171-kbp insert (red bold arrow). Single DNA transfer by the CT system is represented by solid arrows through a light green box. In the mating with the recipient BEST6571 containing a blasticidin S resistance gene (open oval) at the right end of the 196-kbp insert (blue bold arrow), the 171-kbp of transferred DNA recombines with the homologous sequences of the recipient, as indicated by two Xs. Twenty three transcipients with a 345-kbp carrier were selected by markers at both ends as described in Table 1. Bottom: The donor and recipient are reversed. Conversion from BEST6571 to BEST6629 was performed as described above. Thirty three transcipients were selected by both markers as summarized in Table 1. Inserts by I-*Ppo*I digestion^1,2,7,8^ from the indicated *B. subtilis* strains were separated by PFGE. Running conditions: 1% agarose gel with 0.5 × TBE buffer; constant 5 volts/cm; 24 sec pulse time; and 24 hours running time at 14 °C.

As illustrated in Fig. 2, the CT was used to connect two dominos (196 kbp and 171 kbp with a 22 kbp overlap) covering the mouse *jmj* region to yield the 345 kbp product efficiently and accurately.

The original domino strains, BEST6568 (171 kbp, SpcR) and BEST6571 (196 kbp, BSR), which are described in Fig. 2 and Table 1, were converted to CT donors. Two functional elements should be added^9^, namely the sequence *oriT*^110^-*stm* unit, introduced by TF from BEST390 and selected with streptomycin, and pLS20*hyg*, transferred by CT from BEST23124 and selected with hygromycin^9^. Donors are illustrated in Fig. 2 and intermediate strains are listed in Table 1. Mating starts by simple mixing of the stationary donor culture (50 μL) and stationary recipient culture (50 μL) in 2.0 mL fresh LB in a 15 mL-culture tube in the presence of DNase I (final concentration, 3.4 μg per mL). The recipient *B. subtilis* that accepts the DNA transferred from the donor cell by CT is called the transcipient. After incubation at 30 °C for 4 hours, the transcipient was selected on LB plates supplemented with Blasticidin S (BS, 500 μg per mL) and Spectinomycin (Spc, 150 μg per mL). As listed in Table 1, the domino connection by CT yielded 23 transcipients (171 kbp-carrier as donor) and 33 transcipients (196 kbp-carrier as donor) per mL. Representatives of the 23 and 33 transcipients were analyzed by Pulsed Field Gel Electrophoresis (PFGE) as shown in Fig. 2. The production of 345 kbp inserts of the transcipients was confirmed. Sequence comparison of the insert from the donor (171 kbp and 196 kbp kbp) and the transcipient (345 kbp) showed no nucleotide alterations, indicating that the domino connection was achieved accurately.

**Figure 3:**
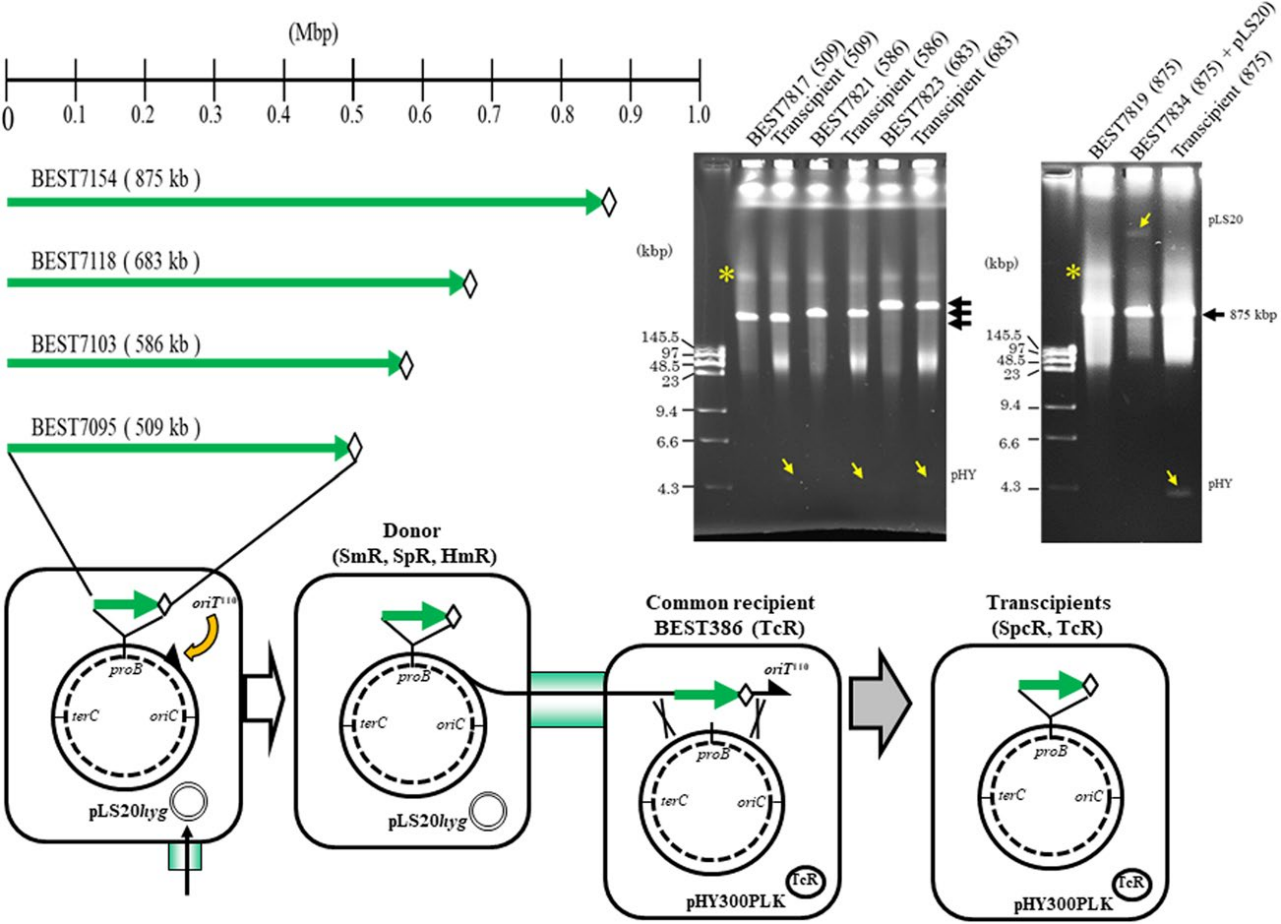
Transfer of DNA as large as 875 kbp by CT. *B. subtilis* strains with the indicated DNA inserts from 509 kbp to 875 kbp, which were reported previously^1^, were converted to the donors listed in Table 1 by the same sequential introduction of the two devices described in Fig. 2. The common recipient BEST386, carrying tetracycline resistance (TcR) by pHY300PLK, is shown and listed in Table 1. The donor marker is Spectinomycin (Spc) + erythromycin (Em), as indicated by an open diamond. Inserts of representative transcipient and donor derivatives (sizes are shown within parentheses) were analyzed by PFGE after complete digestion by I-*Ppo*I. Left gel photo: the positions of the I-*Ppo*I segments of 509, 586, and 683 kbp are indicated by bold horizontal arrows on the right. The same sizes for each donor and transcipient are identified. The size marker lambda DNA ladder (48.5 kbp x n) and *Hin*dIII digests are shown in the left with their sizes in kbp. The plasmid pHY300PLK (4.7 kbp), indicated by pHY on the right, is identified by yellow arrows (↘) in the photo. (_*_) may be produced from *B. subtilis* genome by unidentified I-*Ppo*I star activity (see also Supplementary Fig. 1). Right gel photo: the 875 kbp I-*Ppo*I segment of donor and transcipient is indicated by a bold arrow on the right. The donor BEST7834 carries pLS20*hyg* (66 kbp), as indicated on the right by a yellow arrow (↙) (see also Supplementary Fig. 2). Note that running of circular plasmids >50 kbp on PFGE is difficult to predict^10^. Running conditions: constant 5 volts/cm; 360 sec pulse time; and 17 hours running time at 14 °C.

Next, we investigated whether the CT allows the transmission of large DNA fragments of >200 kb, which was difficult by TF as shown in Fig. 1. We selected four strains carrying synthesized DNA of 509, 586, 683, and 875 kbp, originated from the Cyanobacterium *Synechocystis* spp. PCC6803 genome^1^ as shown in Fig. 3. The original strains shown in Fig. 3 and listed in Table 1 were converted to CT donors by the same method described in Fig. 2. Mating with the common recipient BEST386, which was previously described^9^ and shown in Fig. 3, and selection of transcipients on LB plates supplemented with spectinomycin (Spc,150 μg per mL) and tetracycline (Tc, 10 μg per mL) yielded transcipients (per mL), 125, 86, 140 and 3, as summarized in Table 1. Representatives of each transcipient were analyzed by PFGE, as shown in Fig. 3, which demonstrated the transfer of undisrupted DNA of up to 875 kbp. Sequence comparison of the inserts from donors and transcipients revealed no nucleotide alterations. The efficiency of CT from donors of mouse domino strains to the recipient BEST386 was assessed, and the transcipients were scored as (per mL), 176 (196 kbp), 175 (171 kbp), and 146 (345 kbp), as summarized in Table 1. It seems likely that CT for *B. subtilis* proceeded in a largely size-independent manner, consistent with the Hfr frequency for F-mediated *Eschericha coli*, which is constant throughout the movement of the whole genome^10,11^. The reason for the decrease in the number of transcipients for BEST7834 (875 kbp) remains unclear. A slower growth rate of the donor BEST7834 (875 kbp) (our unpublished observations) may have affected the efficiency of CT.

Regardless of the CT efficiency, the transformants generated by TF, a few from 171, or 196 and none from DNA 345 kb, as summarized in Table 1, are consistent with our previous conclusion^6–8^.

## Discussion

The TF-mediated connection of one domino DNA of 100 kbp requires at least 2 weeks, including the preparation of high quality genomic DNA for TF and selection, followed by screening by antibiotic resistance, and confirmation of insert structures by PFGE. More than 40 weeks was required for the synthesis of 875 kbp DNA by TF^1^. One domino connection by CT is expected to require 1 week including the introduction of two functional elements and the selection of transcipients. Considering that CT-based DNA connection as domino donor and/or recipient can be achieved for up to 683 kbp, the time required for the connection of larger DNA fragments should be dramatically reduced. It should be addressed that the lack of nucleotide alterations caused by CT could minimize the screening for correct transcipients. In addition to the currently established genome synthesis methods using yeast^12,13^, the CT should be a valuable additional tool to accurately edit and synthesize large DNA by *B. subtilis*. The synthesis of existing DNA, even that with a high GC content of up to 70% from *Thermus thermophilus*^8^, chloroplast DNA carrying a long 25-kbp inverse repeat^2^, and mouse genomes with abundant small repeats^7,14,15^ has been demonstrated. In addition to existing DNA, preparation of long DNA *de novo* can be achieved by our method called OGAB^16,17^. Since few nucleotide changes occur during TF^1,16,18^, CT can be used as a daily tool to synthesize DNA fragments of >1000 kbp using long DNA fragments as initial dominos. *B. subtilis* allows the stable synthesis of DNA as large as 3500 kbp^1^ as well as the synthesis of several mouse genome segments;^7,14,15^ therefore, it might be suitable as a workhorse for the Human Genome Wright project^19^.

Lastly, the accurate transfer of 875 kbp DNA inserted in the genome is, to the best of our knowledge, the first and largest exogenous DNA horizontally transferred between *Bacillus* species. The present results and experimental tools might shed light on studies about horizontal DNA transfer^1,3^.

## Electronic supplementary material

 Supplementary Information
