# Supplementary material for: Far rapid synthesis of giant DNA in the Bacillus subtilis genome by a conjugation transfer system
Source: Sci Rep. 2018 Jun 8;8:8792. doi: 10.1038/s41598-018-26987-0 (PMC5993740; doi:10.1038/s41598-018-26987-0)
Supplement: Supplementary file 1 — Supplementary Information [file 41598_2018_26987_MOESM1_ESM.pdf]

Supplementary figures

Far rapid synthesis of giant DNA in the Bacillus subtilis genome by a conjugation transfer system

*Mitsuhiro Itaya, Mitsuru Sato, Miki Hasegawa, Nobuaki Kono, Masaru Tomita, and Shinya Kaneko

Supplementary fig. 1

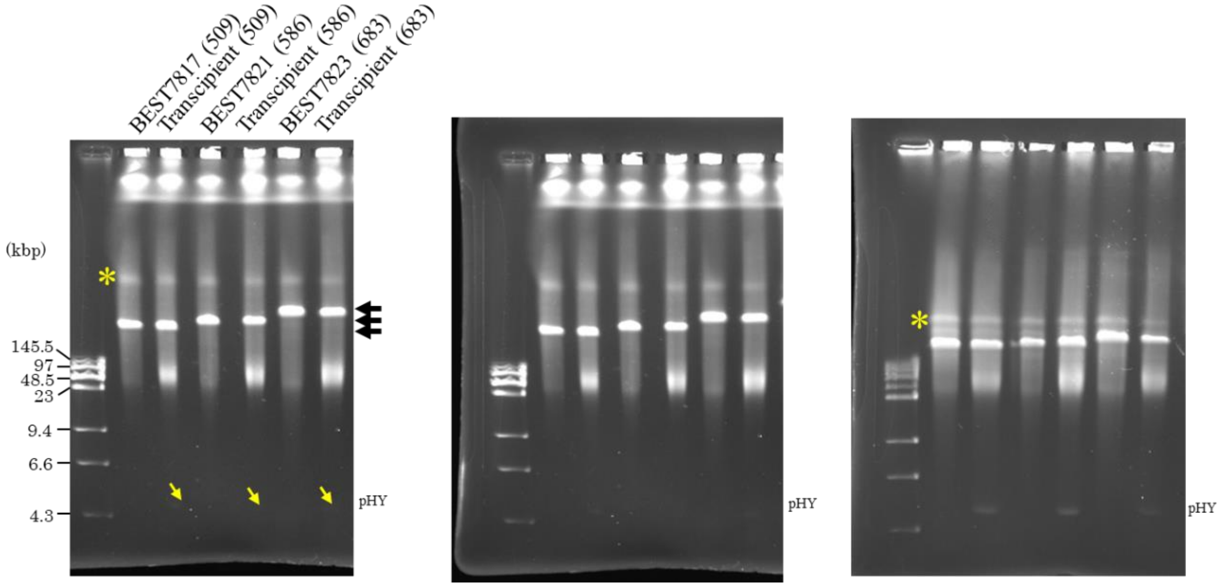

Left: gel photo inserted in Fig. 3. Middle: original gel photo. Right: the same samples run by slightly different running conditions. The pHY position indicated by yellow arrows, very faint in the middle (original) but obviously detectable in the right. The band (*) of the right photo, unrelated to the present result, seemed too strong.

Supplementary fig. 2

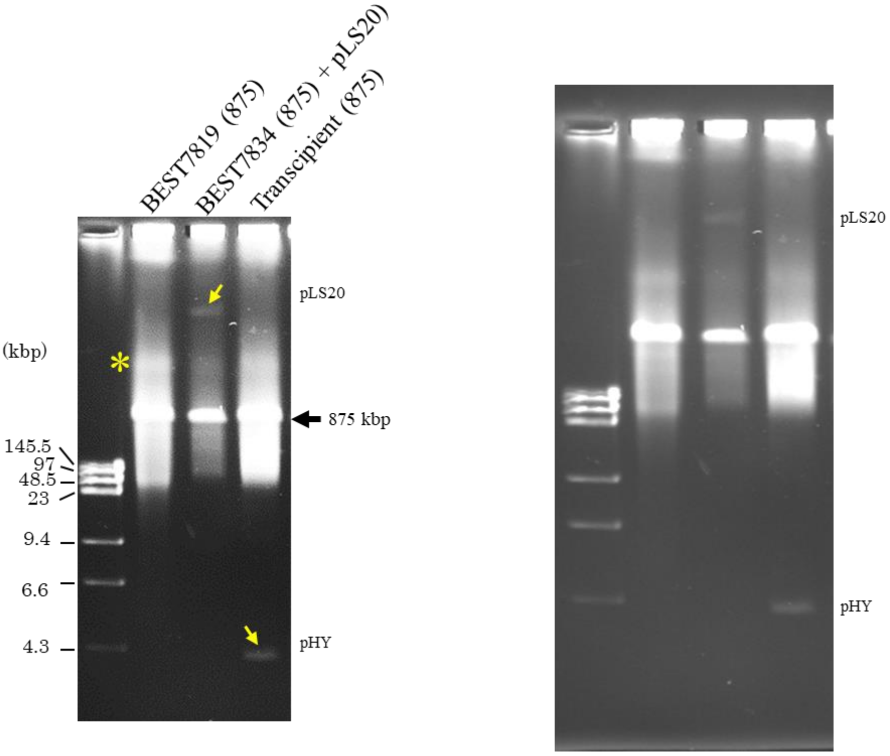

Left: gel photo inserted in Fig. 3. Right: original gel photo. To make the weak pLS20 band of the original photo clearly identifiable, slightly overexposed in the left.
